# Dokumentations- und Behandlungsqualität im Rettungsdienst: eine retrospektive Analyse von Einsatzprotokollen in der Stadt Aachen

**DOI:** 10.1007/s00101-022-01106-x

**Published:** 2022-03-22

**Authors:** Maximilian Klein, Hanna Schröder, Stefan K. Beckers, Christina Borgs, Rolf Rossaint, Marc Felzen

**Affiliations:** 1 Klinik für Anästhesiologie, Medizinische Fakultät, RWTH Aachen University, Uniklinik RWTH Aachen, Aachen, Deutschland; 2 Aachener Institut für Rettungsmedizin und Zivile Sicherheit, Uniklinik RWTH Aachen & Stadt Aachen, Aachen, Deutschland; 3 Ärztliche Leitung Rettungsdienst, Berufsfeuerwehr Aachen, Stadt Aachen, Stolberger Str. 155, 52068 Aachen, Deutschland

**Keywords:** Prähospitale Notfallmedizin, Qualitätssicherung, Notfallsanitäter, Patientensicherheit, Verfahrensanweisungen, Prehospital emergency medicine, Quality assurance, Paramedics, Patient safety, Standard operating procedures

## Abstract

**Hintergrund:**

Der deutsche Rettungsdienst wird jährlich zu ca. 7,3 Mio. Einsätzen alarmiert, welche zu einem Großteil (ca. 59 %) ohne Arzt ablaufen. Da kaum Daten zur Qualität der medizinischen Versorgung und Dokumentation von Rettungsdiensteinsätzen ohne Arzt vorliegen, sollen diese anhand der Einsatzprotokolle im Rahmen dieser Studie überprüft werden.

**Methode:**

Es erfolgte eine retrospektive Analyse von Protokollen der Rettungsdiensteinsätze ohne Arzt aus den Monaten Juni und Juli 2018. Unter Einbezug von Verfahrensanweisungen wurden die Dokumentations- und Behandlungsqualität der Einsätze analysiert. Primäre Endpunkte waren Dokumentationshäufigkeit, Vollständigkeit, die korrekte Notarzt- oder Telenotarztindikationsstellung, die Entwicklung von kritischen Vitalparametern im Einsatzverlauf sowie die mediane Behandlungszeit.

**Ergebnisse:**

Insgesamt wurden 1935 Protokolle ausgewertet. Die Verdachtsdiagnose wurde in 1323 (68,4 %), die Anamnese in 456 (23,6 %), der Erstbefund in 350 (18,1 %) und der Letztbefund in 52 (2,7 %) der Fälle vollständig dokumentiert. Anhand der Dokumentation bestand bei 531 (27 %) Patienten eine Telenotarzt- bzw. Notarztindikation, jedoch kein Arztkontakt. Bei diesen Patienten wurden 410 kritische Vitalparameter im Erstbefund dokumentiert. Von diesen Vitalwerten verbesserten sich 69 (16,8 %); bei 217 (52,9 %) wurde kein Übergabebefund dokumentiert. Die mediane Behandlungsdauer vor Ort war bei Patienten mit eigentlicher Notarztindikation (15:02 min) signifikant länger als bei Patienten ohne Indikation (13:05 min).

**Schlussfolgerung:**

Die Dokumentation der Einsätze ist defizitär. Zudem könnte ein Viertel der Patienten von einem prähospitalen Arztkontakt profitieren. Eine juristisch bedenkliche Übergabedokumentation besteht bei ca. der Hälfte aller Protokolle.

## Abstract (en)

**Background:**

Each year there are 7.3 million emergencies for the German rescue service, trend rising and around 59% of the emergency patients are treated by paramedics only; however, most of the studies focus on physicians, while their practical skills at the scene are rarely necessary. Accordingly, the responsibility for the patient lies with the paramedics most of the time. Their duty is to execute life-saving measures, stabilize the patient for the transport and the regular documentation of the operation. Retrospectively, the emergencies can only be analyzed based on the emergency protocols, which are mostly paper-based and handwritten. That causes an increased effort in the evaluation, which makes studies for the whole country hardly feasible. As of now there are only few data on quality of healthcare and documentation by the paramedics. Both were analyzed in this survey based on the emergency protocols.

**Method:**

A retrospective analysis of emergency protocols from June to July 2018 took place in Aachen, a major German city. A specific feature of Aachen is a 24‑h available emergency physician via telemedicine. The quality of documentation and healthcare was analyzed by including standard operating procedures. Primary endpoints were the frequency of documentation, the achievement of complete documentation, the correct indications for a physician, the development of critical vital signs and the average on-scene time of the ambulance.

**Results:**

Overall, 1935 protocols were analyzed. A complete documentation was achieved in 1323 (68.4%) suspected diagnoses, 456 (23.6%) anamneses, 350 (18.1%) initial and 52 (2.7%) vital signs at handover. Based on the documentation, there were 531 cases (27%) of patients treated by paramedics only, even though a physician would have been indicated. Out of those patients 410 critical initial vital signs were documented of which 69 (16.8%) improved, while there was no documentation of vital signs at handover in 217 (52.9%). Also, there was a significantly prolonged on-scene time for patients with belated indications for an emergency physician with 15:02 min in comparison to 13:05 min for patients without indications.

**Conclusion:**

Deficient documentation was found in multiple cases and several important vital signs for a complete differential diagnosis were missing. Furthermore, a quarter of all patients might have benefited from an emergency physician as they were taken to hospital with no or insufficient treatment, despite standard operating procedures. From a forensic point of view there is an alarmingly incomplete documentation of vital signs at handover. The on-scene time in general was within the predetermined time frame, but can still be reduced in different scenarios. Overall, we recommend strict adherence to the standard operating procedures and algorithms, to remove unnecessary documentation and implement a structured quality assurance. Moreover, the quality of treatment might benefit from the rising number of more specialized paramedics and an increasing use of telemedicine.

Aufgrund zunehmender Notfalleinsätze in Deutschland nimmt auch der Stellenwert der Qualitätssicherung in der Notfallmedizin zu. Es existieren jedoch wenige Daten zu Einsätzen des nichtärztlichen Personals. In der folgenden Studie wird deshalb die Dokumentations- und Behandlungsqualität der durch das Rettungsdienstpersonal durchgeführten Rettungseinsätze überprüft.

## Einleitung

In Deutschland kommt es jährlich zu etwa 7,3 Mio. Notfalleinsätzen mit steigender Tendenz [5]. Der Rettungsdienst hat u. a. die Aufgabe, lebensrettende Maßnahmen durchzuführen, die Transportfähigkeit herzustellen oder aufrechtzuerhalten und den Einsatz regelrecht zu dokumentieren [19]. Studien zur rein rettungsdienstlichen Arbeit und ein strukturiertes Qualitätsmanagement sind jedoch selten. Häufig steht die ärztliche Arbeit im Fokus, obwohl mit knapp 59 % die Mehrheit aller bodengebundenen Rettungseinsätze ohne Arzt abläuft [5], da invasive, einen Arzt vor Ort erfordernde, Maßnahmen selten notwendig sind [10] (z. B. invasive Beatmung 1,4 %, Thoraxpunktion <0,1 % [2]). Zumeist liegt also die Verantwortung für den Patienten beim Rettungsdienstpersonal (RDP). Diese Verantwortung nimmt durch Ausweitung der Befugnisse der Notfallsanitäter weiter zu (§ 2a Notfallsanitätergesetz) [4]. Dementsprechend ist auch die Qualität der Patientenversorgung von der Arbeitsweise des RDP abhängig. Um diese Qualität standardisiert gewährleisten zu können, existieren verbindliche Verfahrensanweisungen (VA) [16]. Ob diese VA routinemäßig befolgt werden, ist mangels Kontrollen bzw. fehlender Studien unklar. Derzeit führt allein das Land Baden-Württemberg über die Stelle zur trägerübergreifenden Qualitätssicherung im Rettungsdienst Baden-Württemberg (SQR-BW) ein landesweites Qualitätsmanagement mit Auswertung aller Rettungsdiensteinsätze durch. Retrospektiv kann die Qualität allenfalls anhand der Dokumentation auf dem Einsatzprotokoll kontrolliert werden. Dieses ist zumeist noch papierbasiert und handschriftlich, wodurch die Auswertung deutlich aufwendiger wird und nicht flächendeckend in Deutschland durchführbar ist. In vorliegender Arbeit soll retrospektiv ein Zeitraum von 2 Monaten als Stichprobe dienen, einen ersten Eindruck der rettungsdienstlichen Arbeit in einem städtischen Rettungsdienstbezirk zu erhalten.

Anhand des Einsatzprotokolls sollen Dokumentations- und Behandlungsqualität erfasst, eine bestehende und durch das RDP nichtberücksichtigte Notarzt(NA)- bzw. Telenotarzt(TNA)-Indikation abgeleitet sowie die Adhärenz zu VA bezüglich dieser Punkte geprüft werden.

## Methode

In dieser retrospektiven Querschnittstudie erfolgte die Auswertung einer Stichprobe von Rettungsdienstprotokollen der Stadt Aachen des Zeitraums Juni und Juli 2018. Die Auswertung erfolgte im Rahmen einer Qualitätsanalyse hinsichtlich Behandlungs- und Dokumentationsqualität. Zur Gewährleistung einer zeitnahen Auswertung der vollständig papierbasierten Dokumentation wurde ein Zeitraum von lediglich 2 Monaten gewählt.

Seit 2014 ist ein TNA 24 h an 7 Tagen der Woche in den Rettungsdienst der Stadt Aachen eingebunden. Dieser wird nicht primär durch die Leitstelle disponiert, sondern bei Notwendigkeit vom RDP selbst konsultiert. Mit der Einführung des TNA wurden viele Meldebilder, welche vor Einführung immer mit NA disponiert wurden, in 2 Ausprägungen, schwer (mit NA) und leicht (ohne NA), gesplittet und dementsprechend disponiert, beispielsweise Hypoglykämie mit Bewusstlosigkeit (NA) und ohne Bewusstlosigkeit (ohne NA). Diese Meldebildsplittung führt durch den restriktiveren Notarzteinsatz zu einer Zunahme der Telekonsultationen.

Die vorliegende Qualitätsanalyse wurde durch die Ethikkommission der Uniklinik RWTH Aachen in der Stellungnahme 357-17 als unbedenklich bewertet.

Die verwendeten Protokolle entsprechen dem Standard der Deutschen Interdisziplinären Vereinigung für Intensiv- und Notfallmedizin (DIVI) und sind laut VA bei jedem Einsatz auszufüllen. Ausgewertet wurden sämtliche Daten des Protokolls, anhängende EKG sowie zugehörige Leitstellendaten. Ausgeschlossen wurden alle Einsätze mit NA- oder TNA-Beteiligung, abgelehnter Behandlung, ohne Transport sowie alle rein psychiatrischen Notfälle.

### Dokumentationsqualität

Primärer Endpunkt war die allgemeine Dokumentationsvollständigkeit von Anamnese (Anamneseschema SAMPLE), Verdachtsdiagnose und Vitalparametern im Erst- und im Letztbefund; diese wurden als unvollständig gewertet, sobald ein zugehöriger Parameter nicht dokumentiert wurde.

Die Auswertung der Verdachtsdiagnose erfolgte anhand des zugehörigen Eingabefeldes, die der Anamnese mittels des Eingabefelds SAMPLE (Anamneseschema) und weiterer Freitextfelder. Als Standardvitalparameter wurden Blutdruck (RR), Herzfrequenz (HF), Sauerstoffsättigung (S_p_O_2_), Atemfrequenz (AF), Temperatur (Temp) und Blutzucker (Bz) festgelegt.

Die Leitsymptomgruppen Angina pectoris, Dyspnoe, Herzrhythmusstörungen, Hypertonie, neurologische Symptome, Schmerzen und Fieber generierten sich aus Freitext- und Eingabefeld. Für jedes Leitsymptom wurde die Dokumentation aller Standardvitalparameter und zusätzlich des EKG als obligat betrachtet.

### Behandlungsqualität

Als Maß für die Behandlungsqualität wurden folgende sekundäre Endpunkte festgelegt:

1. die Häufigkeit des Vorliegens einer NA- bzw. TNA-Indikation, jedoch das Fehlen eines prähospitalen Arztkontaktes (keine Telenotarztkonsultation, kein Notarztnachalarm),
2. die fehlende Verbesserung oder fehlende Dokumentation initial kritischer Vitalwerte bei Übergabe,
3. die Behandlungszeit an der Einsatzstelle.

### Notarzt- bzw. Telenotarztindikation

Vital bedrohte Patienten benötigen ggf. die Fähigkeiten eines NA vor Ort, die Fachkenntnis eines TNA oder Medikamente, die nicht für das RDP freigegeben sind.

Der Rettungsdienst orientiert sich aktuell bei der Nachalarmierung eines NA oder TNA an den Verfahrensanweisungen (VA) des Gemeinsamen Rettungsdienstkompendiums 2020 [16], welche unter Berücksichtigung der S1-Leitlinie Telemedizin [8] erstellt wurden. Einige Kriterien sind in den Protokollen retrospektiv anhand der Dokumentation oft nicht eindeutig nachvollziehbar. Deshalb wurde zur Auswertbarkeit anhand der VA und des durch die SQR-BW modifizierten Münchner NACA-Score (*NACA* National Advisory Committee for Aeronautics) [17] eine NA-/TNA-Indikation mittels der Kriterien in Tab. 1 gestellt.

**Table 1:** 

Kriterium	Bedingung
M‑NACA^a^	≥ 4
Hypertonie	≥ 180 mm Hg systolisch (RRsys) + Symptomatik^b^ mit Endorganschaden vereinbar
Hypertensive Entgleisung	RR ≥ 180/110 mm Hg [28]
Schmerzen	NRS ≥ 5 (Numeric Rating Scale) [9]
Verdacht auf Sepsis	qSOFA Score [3] ≥ 2 oder Verdachtsdiagnose Sepsis
Dyspnoe	+Bronchospasmus, Stridor oder Zyanose

^a^Der modifizierte M‑NACA dient zur Einteilung der Verletzungs- oder Erkrankungsschwere eines Patienten im prähospitalen Bereich [17] und wurde für jedes Einsatzprotokoll durch 2 Experten unabhängig voneinander vergeben und verglichen. Bei unterschiedlichen Angaben wurde aufgerundet. Das RDP vergab den M‑NACA nicht. Ausgeschlossen wurde die Einteilung über die Verletzungsschwere. Weitere Kriterien der Tabelle wurden ergänzend zu Erkrankungsbildern im M‑NACA integriert

^b^Als möglicher Endorganschaden bewertet wurden: starker Kopfschmerz, Dyspnoe, Angina pectoris, akuter Rückenschmerz, Schwindel, Unruhe, Angstzustände, Vigilanzminderung, Wesensveränderung, schwere vegetative Störungen, Nasenbluten, neurologische Ausfälle, Krampfanfall, Übelkeit/Erbrechen

### Übergabedokumentation kritischer Vitalparameter

Die Übergabebefunde der in Tab. 2 aufgeführten Erstbefunde wurden überprüft.

**Table 2:** 

Sauerstoffsättigung (S_p_O_2_)	≤ 90 % [17]
Atemfrequenz (AF)	< 7 oder > 25/min [17]
Herzfrequenz (HF)	< 50 oder > 130/min [17]
NRS	≥ 5 [9]
Hypertonie	RR ≥ 180/110 mm Hg [28], oder RRsys > 180 mm Hg + Endorgansymptomatik

*S*_*p*_*O*_*2*_ Sauerstoffsättigung, *AF* Atemfrequenz, *HF* Herzfrequenz, *NRS* numeric rating scale, *RR* Blutdruck nach Riva-Rocci, *RRsys* systolischer Blutdruck

Diese wurden zum Zeitpunkt der Übergabe als verbessert gewertet bei: S_p_O_2_ > 90 %, AF ≥ 7 und ≤ 25/min, HF ≥ 50 und ≤ 130/min, einem Abfall des mittleren arteriellen Blutdrucks (MAD) um ≥ 20 % und bei einer Reduktion der NRS ≥ 2 Punkte oder auf < 5 [27]. Ansonsten wurden sie als unverändert gewertet.

### Behandlungszeit an der Einsatzstelle

Die mediane Behandlungsdauer an der Einsatzstelle wurde für verschiedene Gruppen verglichen. Eine definitive Zeitvorgabe besteht nicht. Es wurde jedoch ein Intervall von 15 min als Richtwert herangezogen [20].

### Statistische Auswertung

Die deskriptive und quantitative Statistik sowie einige Abbildungen erfolgten über Microsoft Excel für Office 365 MSO (16.0.12527.20612) 32-Bit (Microsoft Corporation, One Microsoft Way, Redmond, WA, 98052-6399, USA). Weitere Abbildungen und Signifikanztestungen erfolgten mittels Mann-Whitney-U-Test auf einem Signifikanzniveau von 5 % mit IBM SPSS Statistics Version 27 (IBM Deutschland GmbH, IBM-Allee 1, 71139 Ehningen, Deutschland). Die Berechnung der Effektstärke erfolgte nach Cohen [7].

## Ergebnisse

### Demografie

**Figure 1:**
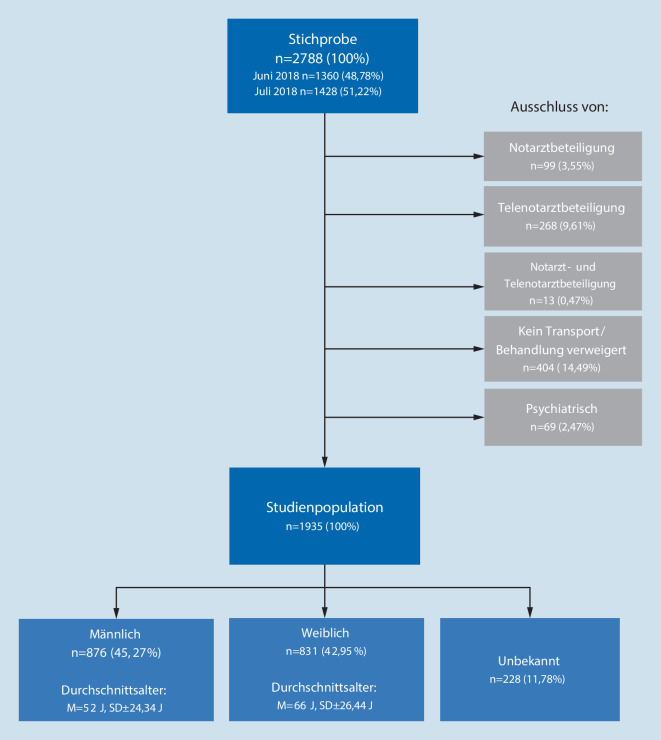

Wie in Abb. 1 dargestellt, wurde in dieser Analyse eine Stichprobe von 2788 Rettungsdienstprotokollen ausgewertet. 853 (30,6 %) Protokolle wurden aufgrund von Beteiligung eines Notarztes bzw. Telenotarztes, der Verweigerung des Transports/der Behandlung oder eines rein psychiatrischen Krankheitsbildes ausgeschlossen. Insgesamt wurden 1935 Rettungsdienstprotokolle ausgewertet. Der Anteil männlicher Patienten betrug 45,27 %, der weiblicher 42,95 % und unbekannten Geschlechtes 11,78 %.

### Dokumentationsqualität

**Figure 2:**
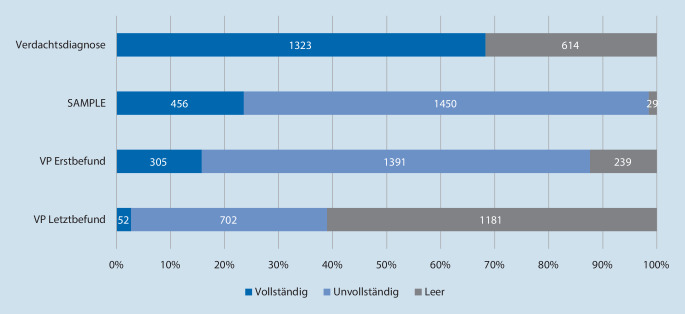

Abb. 2 stellt die Vollständigkeit der Dokumentation dar. Die Verdachtsdiagnose wurde mit 1323 (68,4 %) Fällen am häufigsten, der Übergabebefund mit 52 (2,7 %) Fällen am seltensten dokumentiert.

Tab. 3 zeigt die symptomspezifische Dokumentationshäufigkeit der Vitalparameter. Bei Angina pectoris wurde in 62 (81 %) Fällen und bei Herzrhythmusstörungen in 43 (73 %) Fällen ein EKG dokumentiert. Bei neurologischem Defizit wurde in 247 (52 %) Fällen die Temperatur und in 294 (62 %) der Blutzucker erfasst.

**Table 3:** 

Symptom	Angina pectoris	Dyspnoe	Herzrhythmusstörungen	Hypertonie	Neurologisches Defizit	Schmerzen	Fieber	Studienpopulation
Anzahl (*n*)	77	186	59	81	473	559	37	1935
S_p_O_2_	*77 (100* *%)*	*182 (98* *%)*	*59 (100* *%)*	*81 (100* *%)*	*444 (94* *%)*	499 (89 %)	33 (89 %)	1620 (84 %)
AF	44 (57 %)	118 (63 %)	34 (58 %)	44 (54 %)	255 (54 %)	324 (58 %)	21 (57 %)	**938 (48** **%)**
RR	*76 (99* *%)*	*170 (91* *%)*	*57 (97* *%)*	*80 (99* *%)*	421 (89 %)	441 (79 %)	26 (70 %)	1435 (74 %)
HF	*76 (99* *%)*	*180 (97* *%)*	*59 (100* *%)*	*81 (100* *%)*	*445 (94* *%)*	*505 (90* *%)*	33 (89 %)	1640 (85 %)
EKG	62 (81 %)	100 (54 %)	43 (73 %)	45 (56 %)	**211 (45** **%)**	**151 (27** **%)**	**13 (35** **%)**	**568 (29** **%)**
Bz	**38 (49** **%)**	**68 (37** **%)**	31 (53 %)	44 (54 %)	294 (62 %)	**190 (34** **%)**	**17 (46** **%)**	**742 (38** **%)**
Temp	42 (55 %)	103 (55 %)	34 (58 %)	**38 (47** **%)**	247 (52 %)	**197 (35** **%)**	32 (86 %)	**697 (36** **%)**
Gesamt VP %	77	71	77	73	70	59	67	56

*S*_*p*_*O*_*2*_ Sauerstoffsättigung, *AF* Atemfrequenz, *RR* Blutdruck nach Riva-Rocci, *HF* Herzfrequenz, *EKG* Elektrokardiogramm, *Bz* Blutzucker, *Temp* Temperatur, *VP* Vitalparameter

### Notarzt- bzw. Telenotarztindikation

**Figure 3:**
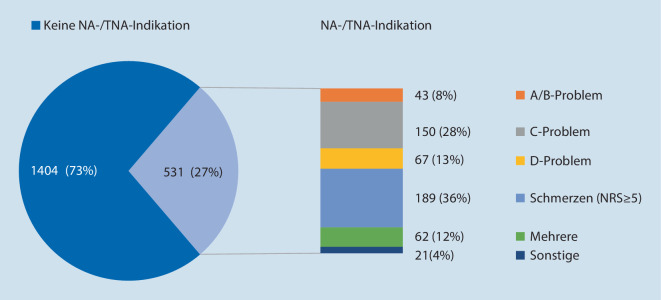

Abb. 3 zeigt Patienten mit und ohne nachträgliche NA-/TNA-Indikation. Es wurden 531 (27 %) Patienten trotz Notarztindikation ohne Kontakt mit einem NA oder TNA transportiert. Den größten Anteil hatten die 189 (36 %) Patienten mit Schmerzen NRS ≥ 5.

### Übergabedokumentation kritischer Erstbefunde

**Figure 4:**
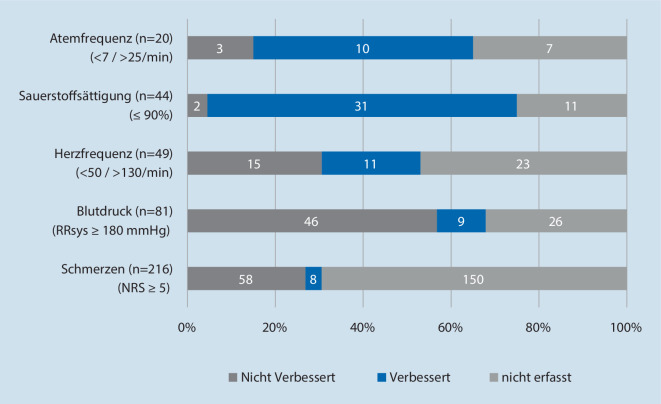

In Abb. 4 wird der Verlauf von kritischen Vitalparametern dargestellt, und wie sich diese zwischen Erst- und Letztbefund verändern. Insgesamt wurden 410 Fälle von kritischen Vitalparametern in den Bereichen Atemfrequenz, Sauerstoffsättigung, Herzfrequenz, Blutdruck und Schmerzen identifiziert und evaluiert.

Davon verbesserten sich insgesamt 69 (16,8 %) Vitalwerte. Bei der Sauerstoffsättigung ist mit 31 Fällen (70,5 %) der Anteil verbesserter Parameter am höchsten.

Zu den 124 (30,2 %) Vitalwerten ohne Verbesserung zählen auch 11 Vitalwerte, bei denen sich eine Verschlechterung (Abweichung des Letztbefunds ≥ 1 zum Erstbefund) wiederfindet (Vitalwert (Anzahl verschlechterte Werte): HF (2), RR (4), Schmerzen (5)).

Der Letztbefund wurde in 217 (52,9 %) Fällen nicht erfasst. Zwischen den Vitalparametern bestehen Unterschiede in der Häufigkeit der Erhebung des Letztbefundes; bei Schmerzen wurde in 66 (30,6 %) Fällen ein weiterer Befund erhoben, bei reduzierter Sauerstoffsättigung in 33 (75 %) Fällen.

### Behandlungszeit an der Einsatzstelle

**Figure 5:**
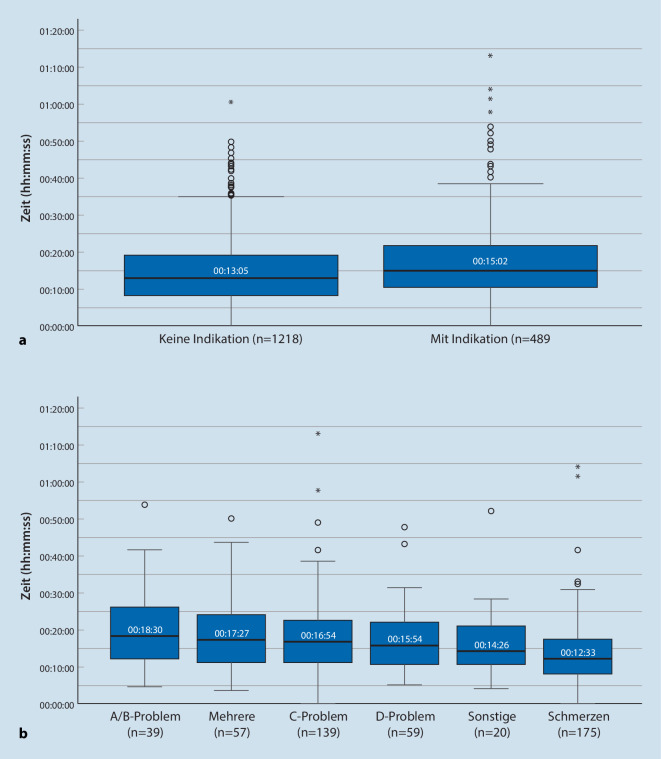

In Abb. 5a wird die Zeit des RTW an der Einsatzstelle dargestellt. Bei Patienten mit NA-/TNA-Indikation (Median (M): 15:02 min, SD ± 09:52 min) besteht eine signifikant längere Verweildauer an der Einsatzstelle, (*p* < 0,05) als ohne NA-/TNA-Indikation (M: 13:05 min, SD ± 08:29 min). Die Effektstärke r = 0,11 entspricht einem schwachen Effekt. Auch bei bestehender Indikation gab es in diesen Einsätzen keinen Arztkontakt.

Für alle Patienten liegt die Behandlungsdauer bei M = 13:40 min.

Abb. 5b zeigt die Behandlungszeiten von Subgruppen mit NA- bzw. TNA-Indikation. Die längste Behandlungsdauer besteht bei Patienten mit Problemen des Atemwegs (*A*) bzw. der Atmung (*B*) (M = 18:30 min). Mit Ausnahme der Patienten mit Schmerzen (M = 12:33 min) und Patienten mit mehreren Diagnosen (M = 14:26 min) liegt die Behandlungsdauer vor Ort bei allen Diagnosen über dem Richtwert von 15 min [20].

## Diskussion

In dieser Arbeit wurden Einsatzprotokolle des Aachener Rettungsdienstes hinsichtlich Dokumentations- und Behandlungsqualität ausgewertet. Während vorhandene Statistiken zum Rettungsdienst über eine digitale Auswertung zwar große Stichproben, jedoch nur definierte Felder erfassen, ermöglichte die manuelle Auswertung in dieser Arbeit die vollständige Protokollevaluation, inklusive Freitextfeldern. Dadurch wurden auswertbare Parameter (beispielsweise Leitsymptome, Anamnese) deutlich erweitert und eine retrospektive Einschätzung des Patientenzustands ermöglicht.

Zusammenfassend zeigte sich neben einer lückenhaften Dokumentation, dass etwa ein Viertel der Patienten von einem prähospitalen Arztkontakt profitieren könnte und auch die Behandlungszeit an der Einsatzstelle optimierungsbedürftig ist.

Hinsichtlich der Dokumentationsvollständigkeit fällt auf, dass die hier vorliegende lückenhafte Dokumentation kein Einzelfall ist, sondern sich ähnlich in anderen Publikationen wiederfindet [12, 26]. Lückenhafte Dokumentation ist demnach keine Seltenheit, vermutlich auch bedingt durch ein hohes Dokumentationsvolumen in einem vergleichsweise kurzen Zeitraum. Dies kann zu durch Informationsverlust bedingten Behandlungsfehlern führen [14]. Deshalb ist die Dokumentation sowohl für die Patientensicherheit als auch aus medikolegaler Sicht von enormer Bedeutung [1]. Ein möglicher Einflussfaktor für die fehlerhafte Dokumentation ist, dass eine abnehmende Erkrankungs‑/Verletzungsschwere, wie sie bei den betrachteten Einsätzen ohne Arzt zumeist vorliegt, mit einer abnehmenden Dokumentationsvollständigkeit einhergehen kann [26]. Aus diesem Grunde sollte vermehrt darauf geachtet werden, bei jedem Einsatz unabhängig von der Erkrankungsschwere mit gleicher Genauigkeit zu arbeiten bzw. zu dokumentieren. Konkret sind regelmäßige Qualitätskontrollen erforderlich, um das konsequente Einhalten von VA mit strukturierter Untersuchung und Anamnese gemäß ABCDE/SAMPLER zu überprüfen. Auch eine digitale Dokumentation mit Pflichtfeldern und Plausibilitätsprüfungen könnte zu einer Verbesserung führen, wie beispielsweise Studien in der Anästhesie zeigten [25]. Zudem sollten redundante Dokumentationselemente abgebaut werden.

Es zeigte sich übereinstimmend mit anderen Studien [18, 26], dass selbst für ein Leitsymptom essenzielle Vitalparameter nicht vollständig erfasst wurden.

Ursächlich könnte die Annahme sein, dass die Erhebung einzelner ausgewählter Vitalparameter zu einer zügigeren und fokussierteren Versorgung des Patienten führt. Die Erhebung aller Standardvitalparameter nimmt jedoch unerheblich mehr Zeit in Anspruch. Außerdem können die vollständige Erhebung der Vitalparameter sowie die konsequente Abarbeitung der entsprechenden VA dabei helfen, Differenzial- oder Nebendiagnosen aufzudecken sowie Fehldiagnosen zu vermeiden [11]. Dies gilt nicht nur für das RDP, sondern gleichermaßen für das ärztliche Personal v. a. bei symptomähnlichen Differenzialdiagnosen [22]. Fehldiagnosen durch Notärzte belaufen sich auf 10–25 % [13, 21]. Trotz fehlender Evidenz lässt sich aufgrund ähnlicher Grundbegebenheiten auch von Fehldiagnosen beim RDP ausgehen.

Bei Überprüfung der Behandlungsqualität zeigt sich, dass bei etwa einem Viertel der Protokolle kein NA/TNA involviert war, obwohl für die Patienten anhand der angewandten Kriterien eine Indikation bestand. Auch im Qualitätsbericht der SQR-BW finden sich, trotz Indikation, Patienten ohne NA-Kontakt wieder [26]. Entscheidend ist, ob das RDP die Notwendigkeit der ärztlichen Intervention erkennt. Individuelle Faktoren, wie Unwissenheit oder ein Hinwegsetzen über VA, sowie die zur Stellung der Notarztindikation einbezogenen Qualitätskriterien des SQR-BW (M-NACA) können eine Rolle spielen. Diese sehen häufiger als in Aachen einen Notarzt vor, ggf. durch unterschiedliche Grundbegebenheiten (kein TNA, Transportzeiten > 20 min [26]).

Die höherfrequente Notwendigkeit eines Arztes könnte z. T. durch einen TNA übernommen werden, ohne einen NA unnötig zu binden. Zur Verbesserung der Versorgungsqualität sollten in den VA bestehende Indikationen streng eingehalten, stichprobenartig kontrolliert, aber auch kritisch reevaluiert und ggf. spezifiziert werden. Des Weiteren könnte ein strukturiertes Feedback der Notaufnahmen zum Patientenzustand zielführend sein. In Zukunft könnte die noch nicht flächendeckende Ausbildung zum Notfallsanitäter zu einer Verbesserung der Patientenversorgung beitragen [4]. Dies ist im Rahmen weiterer Untersuchungen zu prüfen.

Wie auch andere Untersuchungen zeigen [12], wurde selbst bei kritischen Vitalparametern der Übergabebefund häufig nicht dokumentiert. Eine adäquate Verbesserung initial pathologischer Parameter lässt sich dadurch nicht erkennen.

Der Übergabebefund hat eine besondere Bedeutung, da er den Zustand des Patienten an der Schnittstelle zwischen RTW und Notaufnahme schriftlich fixiert. Vor allem bei kritischen Vitalparametern sollten Maßnahmen sowie Veränderungen engmaschig dokumentiert werden, da die Dokumentation bei forensischen Fragestellungen auf diese Weise ent- oder aber belastend sein kann [1]. Werden notwendige Maßnahmen prähospital nicht getroffen, geht die Behandlung auf die zunehmend überlasteten Notaufnahmen über und kann sich dadurch weiter verzögern [6]. Die Transportstrecke ist dabei für die Initiierung einer zügigen Behandlung des Patienten unerheblich. Unter Umständen können prähospitale Maßnahmen durch eine Ausweitung der Kompetenzen der Notfallsanitäter [4] verbessert werden, ein Teil ist jedoch nicht durch fehlende Kompetenzen, sondern durch Motivationsdefizite bedingt. Hinzu kommt der Informationsverlust bei der Patientenübergabe, welcher ebenfalls ein bekanntes Problem ist, welchem durch Etablierung von Übergabestrukturen (z. B. ISOBAR) entgegengewirkt werden kann [23]. Bei nachträglich aufkommenden Fragen hilft nur eine ausreichende Dokumentation auf dem Einsatzprotokoll.

Die durchschnittliche Behandlungszeit vor Ort ist in Aachen kürzer als im bundesweiten Vergleich [24], allerdings ist die Behandlungszeit bei kritischen Patienten signifikant länger als bei nicht-kritischen Patienten.

In der Gruppe der kritischen Patienten kommen unter anderem Tracer-Diagnosen wie Sepsis oder STEMI vor, für welche Vorgaben für die Prähospitalzeit [3, 15] existieren; eine verzögerte Therapie kann hier lebensgefährlich sein. Ursächlich für die verlängerte Behandlung kann z. B. eine fehlende Struktur bzw. Routine in der Patientenversorgung sein. Deshalb könnten beispielsweise Trainings helfen, den strukturierten Einsatzablauf weiter zu optimieren und somit den schnelleren Transport ins Zielkrankenhaus zu optimieren.

### Limitationen der Studie

Bei dieser Auswertung handelt es sich um eine indirekte Analyse der Rettungseinsätze mithilfe der Einsatzprotokolle. Häufig gibt es eine gewisse Diskrepanz zwischen Patientenzustand, durchgeführten Maßnahmen und Dokumentation, zunehmend bei kritischem Patientenzustand. Außerdem ist die handschriftliche Erstellung des Protokolls eine Fehlerquelle. Durch die manuelle Übertragung und Klassifizierung besteht zudem ein „detection bias“ in der Interpretation durch die Auswerter. Durch Auswahl zweier Sommermonate ist das Patientenkollektiv durch saisonale Besonderheiten (beispielsweise Hitzeperioden) nur bedingt repräsentativ. Zudem wurde an einem Rettungsdienststandort mit Option eines TNA ausgewertet. Dadurch ist es unklar, ob sich die Ergebnisse auf die Bundesrepublik Deutschland (BRD) übertragen lassen. Da lediglich Rettungsdienstprotokolle ohne ärztliche Beteiligung ausgewertet wurden, konnten die primären Endpunkte Dokumentationshäufigkeit und -vollständigkeit, die korrekte NA- oder TNA-Indikationsstellung, die Entwicklung von kritischen Vitalparametern im Einsatzverlauf sowie die mediane Behandlungszeit nicht mit Einsätzen, in denen ein NA/TNA nachgefordert wurde, verglichen werden. Bei der Auswertung der kritischen Vitalparameter können chronisch erhöhte oder erniedrigte Parameter das Ergebnis verzerren, indem sie akut veränderte Werte suggerieren.

## Fazit für die Praxis

- Rettungsdienstliche Dokumentation ist in mehreren Bereichen defizitär.
- Die Dokumentation und Behandlung müssen aus Aspekten des Qualitätsmanagements regelmäßig überprüft werden.
- Es existiert eine große Anzahl an nicht adäquat versorgten Patienten, welche durch eine verpflichtende ärztliche Konsultation bzw. Qualitätskontrollen in Form von strukturiertem Feedback der Notaufnahmen verringert werden könnte.
- Vor allem im Umgang mit kritischen Patienten muss die Behandlungszeit an der Einsatzstelle verkürzt werden.
- Weitere multizentrische Studien müssen die Übertragbarkeit auf die BRD überprüfen.
